# Effect of fluoxetine on organ dysfunction and mortality in severe sepsis

**DOI:** 10.1371/journal.pone.0340669

**Published:** 2026-01-21

**Authors:** Islam Abdelaal Abdelmouty Taher, Farouk Kamal Eldin, M. A. Wareth Eissa, Lobna A. Saleh, Osama A. Mohammed, Ahmed S. Doghish, Amr Sobhy Abdel Kway

**Affiliations:** 1 Department of Anesthesia, Intensive Care and Pain Management, Faculty of Medicine, Ain Shams University, Cairo, Egypt; 2 Department of Clinical Pharmacology, Faculty of Medicine, Ain Shams University, Cairo, Egypt; 3 Department of Pharmacology and Toxicology, College of Pharmacy, Taif University, Taif, Saudi Arabia; 4 Department of Pharmacology, College of Medicine, University of Bisha, Bisha, Saudi Arabia; 5 Department of Biochemistry, Faculty of Pharmacy, Badr University in Cairo (BUC), Badr City, Cairo, Egypt; 6 Biochemistry and Molecular Biology Department, Faculty of Pharmacy (Boys), Al-Azhar University, Nasr City, Cairo, Egypt; Kyung Hee University, KOREA, REPUBLIC OF

## Abstract

**Introduction:**

Sepsis is a leading cause of morbidity and mortality in intensive care units, characterized by a dysregulated host response to infection. Recent evidence suggests fluoxetine, a selective serotonin reuptake inhibitor, may exert immunometabolic effects beneficial in sepsis. The aim of this study is to evaluate the effect of fluoxetine on vasopressor duration, organ dysfunction, inflammatory markers, and mortality in adult patients with severe sepsis.

**Materials and methods:**

In this single-center, randomized, double-blind, placebo-controlled trial conducted at Ain Shams University Hospitals (December 2024–June 2025), 46 patients with severe sepsis were randomized 1:1 to receive either fluoxetine (40 mg/day) or placebo in addition to standard sepsis care. The primary outcome was vasopressor duration. Secondary outcomes included Sequential Organ Failure Assessment (SOFA) scores, inflammatory biomarkers (CRP, TNF-α, IL-1, procalcitonin), lactate levels, ICU length of stay, and 28-day mortality.

**Results:**

Fluoxetine significantly reduced vasopressor duration (6.2 ± 0.4 vs. 7.9 ± 0.8 days; p < 0.001), ICU stay (15.9 ± 1.6 vs. 17.1 ± 1.1 days; p = 0.005), and inflammatory markers by day 7, including TNF-α, IL-1, CRP, and procalcitonin (all p < 0.05). SOFA and APACHE II scores were also lower in the fluoxetine group on days 7 and 10. No significant difference in 28-day mortality was observed (8.7% vs. 17.4%; p = 0.381).

**Conclusions:**

Fluoxetine as adjunctive therapy in severe sepsis may reduce vasopressor dependence, attenuate inflammation, and shorten ICU stay without increasing adverse effects. Its mortality benefit remains uncertain and warrants further investigation.

## Introduction

Sepsis and septic shock pose a significant and escalating global challenge for healthcare providers due to their rising incidence and the complex pathophysiological, molecular, genetic, and clinical factors involved. Since the annual burden of sepsis in high-income countries is rising, with a mortality of 40%. Despite these figures from industrialized countries, the largest part of the global sepsis burden occurs in middle and low-income countries [1].

Severe sepsis is characterized by sepsis accompanied by organ dysfunction, hypoperfusion, or hypotension. Perfusion issues can manifest as lactic acidosis, oliguria, or changes in mental status, among others. In 2016, the Surviving Sepsis Campaign defined sepsis as a life-threatening organ dysfunction resulting from an unregulated host response to infection [2].

In addition to fluid resuscitation, vasopressor therapy is a fundamental treatment of septic shock-induced hypotension as it aims at correcting the vascular tone depression and then at improving organ perfusion pressure. Experts’ recommendations currently position norepinephrine (NE) as the first-line vasopressor in septic shock. One problem with the use of vasopressors is the risk of side effects (like Cardiac arrhythmia, Peripheral ischemia, Inadvertent immunomodulation) and the ensuing need for intensive care management, which is costly. Studies have demonstrated that vasopressor use can be associated with specific side effects, and prolonged use may be associated with mortality [3].

Fluoxetine, an antidepressant classified as a selective serotonin reuptake inhibitor (SSRI), increases levels of serotonin, a natural chemical in the brain [4]. SSRIs are now acknowledged for their extensive peripheral effects, including the regulation of immune and metabolic processes [5,6]. Additionally, studies have demonstrated that SSRIs can provide protection against sepsis in animal models [7] and enhance outcomes in patients infected with SARS-CoV-2 [8].

This study was conducted to evaluate the efficiency of fluoxetine as an adjuvant therapy in the treatment of severe sepsis and its effect on multiple organ dysfunction and mortality in septic patients.

## Materials and methods

### Ethical statement

The study protocol received approval from the Research Ethics Committee, Faculty of Medicine, Ain Shams University, and was prospectively registered at the Pan African Clinical Trial Registry (PACTR202412745051519). The study complies with all regulations, and written informed consent was obtained from all participants. The trial adhered to the CONSORT 2010 guidelines and the Declaration of Helsinki. Informed consent was obtained from the legal surrogates of all participants before enrolment. Informed consent was obtained from each participant prior to their participation in the investigation.

### Study design and patient selection

A single-centre, randomized, double-blind, placebo-controlled trial was conducted in the mixed medical-surgical Intensive Care Unit (ICU) of Ain Shams University Hospitals, Cairo, Egypt, from 13/12/2024–02/06/2025.

Eligible participants were adults aged 18–65 years with a body mass index (BMI) between 30 and 45 kg/m^2^, diagnosed with severe sepsis based on Sepsis Surviving Campaign (SSC) 2016, which required the presence of a suspected or confirmed infection and a Sequential Organ Failure Assessment (SOFA) score of ≥2. Additional criteria for inclusion included hypotension (mean arterial pressure <65 mmHg requiring vasopressors) or hyperlactatemia (lactate >2 mmol/L). Exclusion criteria included an APACHE II score greater than 25, liver cirrhosis, end-stage renal disease (estimated glomerular filtration rate <15 mL/min/1.73 m^2^), QTc interval >500 ms, pregnancy, fluoxetine allergy, gastro intestinal dysfunction, malignancy, active haemorrhage, burns, chronic use of corticosteroids (>1 mg/kg/day prednisone equivalent), and conditions such as chronic use of antidepressants (SSRIs, SNRIs, tricyclic antidepressants), monoamine oxidase inhibitors, or QT-prolonging drugs

### Interventions

Participants were randomized in a 1:1 ratio to receive either the standard care treatment or the fluoxetine intervention. Randomization was performed using computer-generated block randomization (block size = 4), stratified by infection source (pneumonia, intra-abdominal, urinary, or soft tissue). Allocation concealment was ensured through sequentially numbered, opaque, tamper-evident envelopes prepared by an independent ICU pharmacist. Both the participants and treating clinicians, as well as outcome assessors and statisticians, were blinded to group assignments. Fluoxetine and placebo were identically packaged by the hospital pharmacy to maintain blinding.

**The control group** received standard care based on the Surviving Sepsis Campaign (SSC) guidelines, including empiric antibiotics (piperacillin-tazobactam or meropenem) administered within one hour of diagnosis, intravenous fluid resuscitation (30 mL/kg crystalloids), and norepinephrine for vasopressor support to maintain a mean arterial pressure ≥65 mmHg. The **fluoxetine group** received standard care as described above, plus fluoxetine 40 mg (Prozac 20 mg capsules, Eli Lilly, Indianapolis, Indiana, USA), administered orally or via a nasogastric tube fluoxetine syrup (fluoxetine oral solution USP (20 mg/5 ml), Pai pharmaceutical associates, Greenville, south Carolina, USA) for up to 28 days, or until ICU discharge or death, whichever occurred first. TA protocol for enteral administration was followed, with gastric residual volume (GRV) monitored every 4 hours; if GRV exceeded 300 mL, intravenous metoclopramide (10 mg) was administered. Enteral feeding was paused if GRV exceeded 500 mL. To confirm the pharmacokinetics of fluoxetine absorption, plasma fluoxetine and its active metabolite, norfluoxetine, were measured in a subgroup of 10 patients on days 3 and 7.

### Outcomes

The primary outcome of the study was the duration of vasopressor or inotrope dependency, measured in hours from randomization until successful discontinuation of these agents. Secondary outcomes included inflammatory markers (C-reactive protein, procalcitonin, tumour necrosis factor-alpha, and interleukin-1) measured on days 1, 3, 7, and 14, as well as the number of ICU-free days within 28 days, all-cause 28-day mortality, daily SOFA scores from days 1–7, and the incidence of adverse events such as QTc prolongation, arrhythmias, and gastrointestinal intolerance.

### Sample size calculation

The sample size was based on a meta-analysis by Rygård et al. [9], which reported a mean reduction in shock duration of −1.52 days with corticosteroids compared to placebo (95% confidence interval: −1.71 to −1.32). Using this effect size, a two-sided alpha of 0.05, and 80% power, we calculated that 23 patients per group (46 total) would be sufficient to detect a statistically significant difference.

### Randomization and blinding

**Fig 1:**
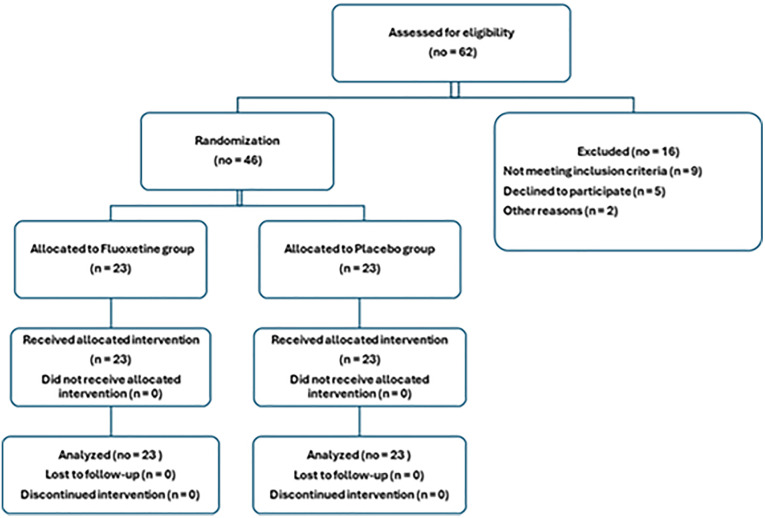
CONSORT 2025 flow diagram. Enrolled patients in a randomized controlled clinical trial on the effect of fluoxetine on organ dysfunction and mortality in patients with severe sepsis.

Randomization was performed using computer-generated block randomization (block size = 4), stratified by sepsis source (pneumonia, intra-abdominal, urinary, soft tissue). Allocation concealment was ensured by sequentially numbered, opaque, tamper-evident envelopes prepared by an independent ICU pharmacist. Both participants and clinicians, as well as outcome assessors and statisticians, were blinded to group assignment. Fluoxetine and placebo were identically packaged by the hospital pharmacy to maintain blinding. Fig 1 shows a consensus flowchart (Flow diagram of the progress through the phases of a randomised trial of two groups (that is, enrolment, intervention allocation, follow-up, and data analysis)).

### Statistical analysis

Statistical analysis was performed using SPSS v29.0 (IBM Corp., Armonk, NY) and R 4.3.1. Continuous variables were summarized as mean ± SD or median (IQR) and compared using the Student’s t-test or Mann–Whitney U-test. Categorical variables were compared using the χ² or Fisher’s exact test. The primary outcome (vasopressor duration) was analysed using a mixed-effects linear regression model adjusted for baseline APACHE II and SOFA scores. Secondary longitudinal outcomes were analysed using repeated-measures ANOVA or mixed-effects models. Kaplan–Meier survival curves with log-rank tests were used to compare 28-day mortality. Vasopressor duration and ICU stay were analysed as continuous outcomes because all observations were complete with no censoring. No adjustment for multiple comparisons was applied; secondary outcomes are interpreted as exploratory. Missing data (<10%), due mainly to early discharge or death, were handled using multiple imputation by chained equations (20 datasets). Analyses were performed in each imputed dataset and pooled using standard combination rules. Statistical significance was defined as P < 0.05 (two-sided).

## Results

**Table 1** compares baseline characteristics between the Fluoxetine and Control groups. No statistically significant differences were observed in gender distribution (p = 0.767), age (p = 0.114), weight (p = 0.804), or cause of sepsis (p = 0.937), suggesting balanced randomization. Both groups had similar proportions of abdominal sepsis, UTIs, and pneumonia as sepsis

**Table 1:** Baseline demographic and clinical characteristics of patients in the fluoxetine and control groups.

		Fluoxetine		Control		
		Mean ±SD	95% CI	Mean ±SD	95% CI	P-value
Gender	Male	12	52%	11	48%	0.767
	Female	11	48%	12	52%	
Age (years)		47 ± 8.98	(43.33 - 50.67)	50.61 ± 5.88	(48.21 - 53.01)	0.114
Weight (kg)		92.74 ± 9.55	(88.84 - 96.64)	92.09 ± 8.08	(88.79 - 95.39)	0.804
cause of sepsis	Abdominal Sepsis	9	39%	10	43%	0.937
	UTI	7	30%	7	30%	
	Pneumonia	7	30%	6	26%	

SD = standard deviation; CI = confidence interval; UTI = urinary tract infection. Data presented as mean ± SD (95% CI) for continuous variables and frequency (%) for categorical variables. P-values calculated using independent t-tests (age, weight) and chi-square tests (gender, cause of sepsis). Statistical significance set at P-value <0.05.

etiologies. The lack of baseline differences supports the internal validity of subsequent outcome comparisons. However, the small sample size (n = 23 per group) may limit the power to detect subtle differences. Further studies with larger cohorts are warranted to confirm these findings.

**Table 2** summarizes the temporal evolution of organ dysfunction scores (SOFA and APACHE II) and inflammatory biomarkers (lactate, CRP, TNF-α, IL-1, and procalcitonin) in both study arms across five predefined assessment points. Each cell presents the mean ± standard deviation and the corresponding 95% confidence interval, enabling direct comparison of the magnitude and precision of estimates at each time point. Between-group comparisons at individual time points were evaluated using independent t-tests. To account for the repeated-measures structure of the dataset, a full repeated-measures ANOVA (RM-ANOVA) model was applied to each variable. The “RM-ANOVA P-value” represents the main effect of treatment group, reflecting whether the fluoxetine and control cohorts differ in overall levels of the outcome when averaged across all time points. The “RM-ANOVA P-Time (GG)” value quantifies the within-subject effect of time, corrected using the Greenhouse–Geisser adjustment to address violations of sphericity. This statistic tests whether the biomarker or score changes significantly over the clinical course, regardless of treatment allocation.

**Table 2:** Longitudinal clinical and biomarker outcomes in the fluoxetine and control groups.

		Mean ±SD 95% CI	Mean ±SD 95% CI	t-test P-value	RM-ANOVA P-value	RM-ANOVA P-Time (GG)	RM-ANOVA P-Group×Time (GG)	εGG
SOFA	Baseline	**8.65 ± 1.94** 95% CI (7.86–9.44)	**8.96 ± 2.12** 95% CI (8.09–9.83)	0.615	0.002*	<0.001*	0.015*	0.447
	Day 3	**6.57 ± 1.93** 95% CI (5.78–7.36)	**6.35 ± 1.61** 95% CI (5.69–7.01)	0.680				
	Day 7	**3.78 ± 1.48** 95% CI (3.18–4.38)	**4.7 ± 1.55** 95% CI (4.07–5.33)	0.047*				
	Day 10	**2.52 ± 0.9** 95% CI (2.15–2.89)	**3.22 ± 1.04** 95% CI (2.79–3.65)	0.046*				
	Day 14	**1.78 ± 0.85** 95% CI (1.43–2.13)	**2.43 ± 0.84** 95% CI (2.09–2.77)	0.177				
APACHE II	Baseline	**22 ± 4.61** 95% CI (20.12–23.88)	**20.61 ± 5.65** 95% CI (18.3–22.92)	0.365	0.049*	<0.001*	<0.001*	0.411
	Day 3	**16.22 ± 3.84** 95% CI (14.65–17.79)	**15.78 ± 4.64** 95% CI (13.88–17.68)	0.731				
	Day 7	**8.96 ± 3.7** 95% CI (7.45–10.47)	**11.35 ± 3.42** 95% CI (9.95–12.75)	0.028*				
	Day 10	**6.74 ± 3.19** 95% CI (5.44–8.04)	**8.26 ± 2.3** 95% CI (7.32–9.2)	0.048*				
	Day 14	**6.0 ± 3.15** 95% CI (4.71–7.29)	**6.09 ± 1.86** 95% CI (5.33–6.85)	0.910				
Lactate (mmol/L)	Baseline	**8.07 ± 1.81** 95% CI (7.33–8.81)	**8.47 ± 1.72** 95% CI (7.77–9.17)	0.669	0.001*	<0.001*	0.001*	0.508
	Day 3	**6.14 ± 1.48** 95% CI (5.54–6.74)	**6.72 ± 1.59** 95% CI (6.07–7.37)	0.212				
	Day 7	**3.63 ± 1.79** 95% CI (2.9–4.36)	**4.87 ± 1.21** 95% CI (4.38–5.36)	0.008*				
	Day 10	**2.31 ± 1.15** 95% CI (1.84–2.78)	**3.47 ± 1.06** 95% CI (3.04–3.9)	0.038*				
	Day 14	**1.82 ± 0.8** 95% CI (1.49–2.15)	**2.68 ± 0.79** 95% CI (2.36–3)	0.462				
CRP (mg/dl)	Baseline	**103.82 ± 39.41** 95% CI (87.71–119.93)	**108.93 ± 41.18** (92.1 - 125.76)	0.669	0.405	<0.001*	0.065	0.326
	Day 3	**73.49 ± 29.8** 95% CI (61.31–85.67)	**72.95 ± 28.42** 95% CI (61.34–84.56)	0.950				
	Day 7	**41.17 ± 22.8** 95% CI (31.85–50.49)	**49 ± 15.87** 95% CI (42.51–55.49)	0.004*				
	Day 10	**20.52 ± 14.13** 95% CI (14.75–26.29)	**35.45 ± 11.65** 95% CI (30.69–40.21)	0.013*				
	Day 14	**19.16 ± 10.6** 95% CI (14.83–23.49)	**26.32 ± 8.58** 95% CI (22.81–29.83)	0.272				
TNF-a (pg/ml)	Baseline	**96.44 ± 9.89** 95% CI (92.4–100.48)	**97.96 ± 9.95** 95% CI (93.89–102.03)	0.606	<0.001*	<0.001*	<0.001*	0.464
	Day 3	**71.99 ± 14.37** 95% CI (66.12–77.86)	**71.92 ± 12.3** 95% CI (66.89–76.95)	0.986				
	Day 7	35.78 ± 15.13 95% CI (29.6–41.96)	51.13 ± 12.06 95% CI (46.2–56.06)	0.000*				
	Day 10	**26.62 ± 12.54** 95% CI (21.5–31.74)	**36.27 ± 8.35** 95% CI (32.86–39.68)	0.004*				
	Day 14	**22.67 ± 10.5** 95% CI (18.38–26.96)	**25.49 ± 8.2** 95% CI (22.14–28.84)	0.316				
IL-1 (pg/ml)	Baseline	**88.66 ± 8.75** 95% CI (85.08–92.24)	**88.33 ± 6.07** 95% CI (85.85–90.81)	0.882	<0.001*	<0.001*	<0.001*	0.467
	Day 3	**60.25 ± 13.11** 95% CI (54.89–65.61)	**63.88 ± 10.52** 95% CI (59.58–68.18)	0.305				
	Day 7	**31.13 ± 13.75** 95% CI (25.51–36.75)	**45.16 ± 10.31** 95% CI (40.95–49.37)	0.000*				
	Day 10	**16.93 ± 6.13** 95% CI (14.42–19.44)	**32.36 ± 8.23** 95% CI (29–35.72)	0.015*				
	Day 14	**17.17 ± 6.79** 95% CI (14.4–19.94)	**24.17 ± 6.94** 95% CI (21.33–27.01)	0.144				
Procalcitonin (pg/dl)	Baseline	**9.97 ± 2.31** 95% CI (9.03–10.91)	**10.43 ± 2.4** 95% CI (9.45–11.41)	0.506	0.005*	<0.001*	0.092	0.353
	Day 3	**7.33 ± 1.78** 95% CI (6.6–8.06)	**8.04 ± 2.26** 95% CI (7.12–8.96)	0.246				
	Day 7	**4.5 ± 1.59** 95% CI (3.85–5.15)	**5.89 ± 1.79** 95% CI (5.16–6.62)	0.008*				
	Day 10	**2.62 ± 0.67** 95% CI (2.35–2.89)	**4.3 ± 1.37** 95% CI (3.74–4.86)	0.014*				
	Day 14	**2.18 ± 0.74** 95% CI (1.88–2.48)	**3.24 ± 1.25** 95% CI (2.73–3.75)	0.216				

SD = standard deviation; CI = confidence interval; SOFA = Sequential Organ Failure Assessment; APACHE II = Acute Physiology and Chronic Health Evaluation II; CRP = C-reactive protein; TNF-α = tumor necrosis factor-alpha; IL-1 = interleukin-1. Data presented as mean ± SD (95% CI); RM-ANOVA = Repeated-measures analysis of variance; P-Group = between-group main effect; P-Time (GG) = within-subject time effect corrected using the Greenhouse–Geisser adjustment; P-Group × Time (GG) = group-by-time interaction with Greenhouse–Geisser correction; εGG = Greenhouse–Geisser epsilon (measure of sphericity violation); P-values calculated using-test then repeated-measures ANOVA or mixed-effects models for longitudinal comparisons. (*) Denotes statistical significance-value <0.05.

The “RM-ANOVA P-Group × Time (GG)” interaction term evaluates whether the trajectory of improvement differs between groups—this is the key indicator of whether fluoxetine alters the rate or pattern of physiological recovery over time. Finally, the Greenhouse–Geisser epsilon (εGG) is reported for transparency regarding the extent of sphericity violation; lower εGG values indicate increased departure from the sphericity assumption and justify greater correction of degrees of freedom in the time-related tests. The combination of these analyses demonstrates that fluoxetine not only reduces several markers of systemic inflammation more rapidly but also significantly accelerates improvement in SOFA, APACHE II, lactate, TNF-α, IL-1, and procalcitonin, with multiple significant Group × Time interactions confirming diverging recovery trajectories between the two study arms beginning as early as Day 7.

**Table 3** compares clinical outcomes and interventions between the Fluoxetine and Control groups. The Fluoxetine group had a significantly shorter duration of vasopressor use among patients requiring vasopressors (6.22 vs. 7.93 days, p < 0.001), suggesting potential hemodynamic benefits. Significant differences were observed in ICU length of stay (p = 0.005), survival rates (48% vs. 35%, p = 0.369), or need for haemodialysis (p = 0.474) or mechanical ventilation (p = 1.000). Notably, the numerical reduction in mortality (8.7% vs. 17.4%) in the Fluoxetine group did not reach statistical significance, possibly due to limited power. The small sample size and variability in outcomes (e.g., wide SD for MV duration in Fluoxetine) highlight the need for larger studies to confirm these trends

**Table 3:** Clinical outcomes and interventions in the fluoxetine and control groups.

		Fluoxetine		Control		
		Mean ±SD	95% CI	Mean ±SD	95% CI	P-value
length of ICU stays		15.91 ± 1.56	(15.27 - 16.55)	17.09 ± 1.12	(16.63 - 17.55)	0.005 *
Vasopressors need	Yes	9	39%	14	61%	0.14
	No	14	61%	9	39%	
Vasopressor duration	for patients who needed a vasopressor	6.22 ± 0.44	(6.04 - 6.4)	7.93 ± 0.83	(7.59 - 8.27)	<0.001*
HDx need	Yes	4	17%	6	26%	0.474
	No	19	83%	17	74%	
MV need	Yes	10	43%	10	43%	1
	No	13	57%	13	57%	
MV duration		2.83 ± 3.46	(1.42 - 4.24)	6.22 ± 0.85	(5.87 - 6.57)	0.565
Survival	Yes	21	91.3%	19	82.6%	0.381
	No	2	8.7%	4	17.4%	

ICU = intensive care unit; HDx = hemodialysis; MV = mechanical ventilation; SD = standard deviation; CI = confidence interval. Data presented as mean ± SD (95% CI) for continuous variables and frequency (%) for categorical variables. P-values calculated using independent t-tests (continuous variables) and chi-square/Fisher’s exact tests (categorical variables). *Denotes statistical significance, P-value < 0.05.

**Fig 2:**
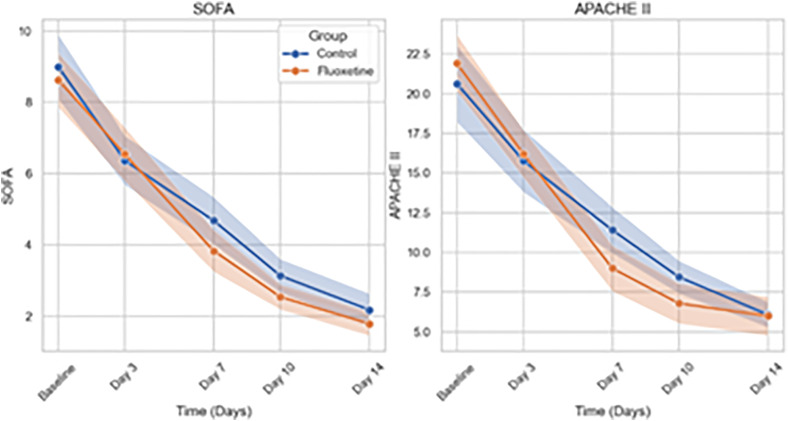
Temporal Changes in SOFA and APACHE II Scores Over 14 Days.

In **Fig 2**, line plots represent the mean (solid lines) and 95% confidence intervals (shaded areas) of Sequential Organ Failure Assessment (SOFA) scores and Acute Physiology and Chronic Health Evaluation II (APACHE II) scores over a 14-day period for Fluoxetine and Control groups. Mixed-effects linear regression models with random intercepts were used to assess the effect of treatment group, time, and their interaction. A significant group-by-time interaction was observed for SOFA scores (p = 0.037), indicating a greater reduction in the Fluoxetine group. No significant interaction was observed for APACHE II scores (p = 0.459).

**Fig 3:**
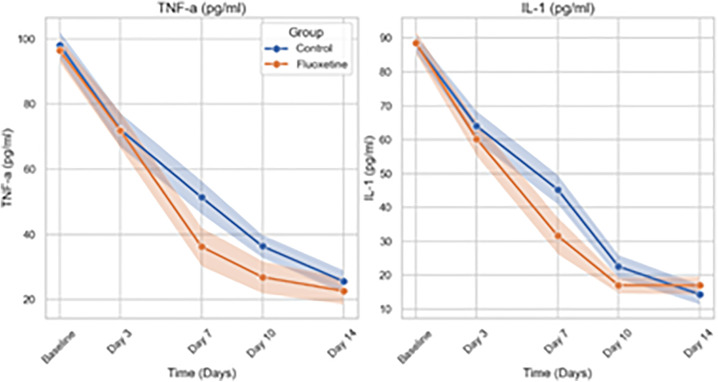
Temporal Changes in TNF-α and IL-1 Levels Over 14 Days.

In **Fig 3**, line plots show the mean (solid lines) and 95% confidence intervals (shaded areas) of tumor necrosis factor-alpha (TNF-α) and interleukin-1 (IL-1) serum concentrations over a 14-day period in the Fluoxetine and Control groups. Both biomarkers showed significant declines over time (p < 0.001); however, the group-by-time interactions were not statistically significant (p = 0.163 for TNF-α, p = 0.074 for IL-1), suggesting comparable trajectories between groups.

**Fig 4:**
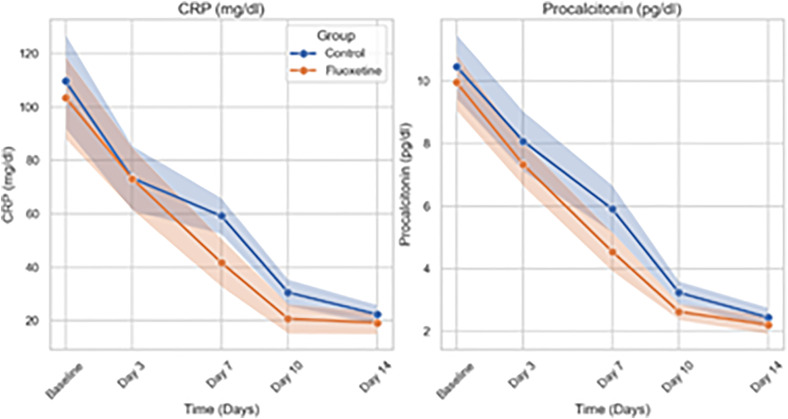
Temporal Changes in CRP and Procalcitonin Levels Over 14 Days.

In **Fig 4**, line plots depict the mean (solid lines) and 95% confidence intervals (shaded areas) of C-reactive protein (CRP) and procalcitonin concentrations over a 14-day period in the Fluoxetine and Control groups. Both inflammatory biomarkers decreased significantly over time (p < 0.001). No significant group-by-time interactions were observed for CRP (p = 0.237) or procalcitonin (p = 0.136), indicating similar rates of inflammatory resolution across groups

Mixed-effects linear regression analysis demonstrated significant reductions over time in SOFA and APACHE II scores and inflammatory biomarkers (TNF-α, IL-1, CRP, and procalcitonin) across all patients (p < 0.001 for time effects). A significant group-by-time interaction was observed for SOFA scores (Estimate = −0.10; 95% CI: −0.20 to −0.01; p = 0.037), indicating a faster decline in the Fluoxetine group compared to controls, whereas no significant interaction was found for APACHE II scores (p = 0.459). Lactate levels similarly showed a significant group-by-time interaction favoring Fluoxetine (p = 0.035). In contrast, although TNF-α, IL-1, CRP, and procalcitonin levels declined significantly over time, no significant group-by-time interactions were observed (p > 0.05 for all), suggesting similar inflammatory resolution rates between groups. These results suggest that fluoxetine may accelerate the improvement of organ dysfunction and metabolic recovery, while the resolution of systemic inflammation followed comparable trajectories in both groups.

**Fig 5:**
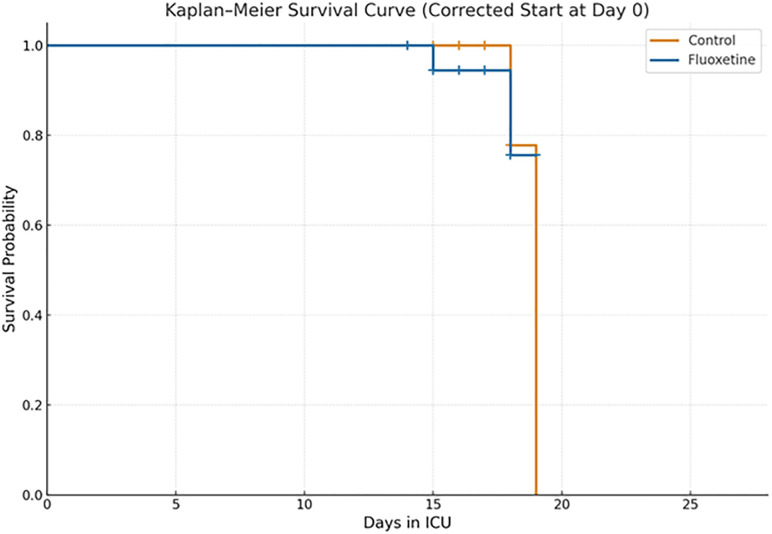
Kaplan–Meier survival estimates for 28-day mortality comparing the fluoxetine and control groups.

**Fig 5** illustrates the Kaplan–Meier survival estimates for 28-day mortality comparing the fluoxetine and control groups. Both survival curves remained nearly superimposed throughout the observation period, with only a small number of events occurring in either arm. There was no statistically significant difference in 28-day survival between groups, as demonstrated by the log-rank test (P = 0.863). Overall, the analysis indicates comparable short-term survival between fluoxetine and control arms, with no evidence of divergence in risk over time.

## Discussion

Sepsis remains a formidable challenge in critical care medicine, characterized by a dysregulated host response to infection leading to life-threatening organ dysfunction and significant mortality worldwide [2,10]^**.**^ Despite advances in antimicrobial therapy and supportive care, treatment options are often limited, particularly concerning the host’s detrimental inflammatory and metabolic responses. Traditional approaches targeting specific pro-inflammatory cytokines have largely failed in clinical trials, potentially due to issues with timing, patient heterogeneity, and the risk of immunosuppression [11,12]. This underscores the urgent need for novel host-directed therapies that can modulate the complex pathophysiology of sepsis, aiming not just to control the pathogen but also to mitigate host-inflicted damage and restore physiological homeostasis [13].

In the search for such therapies, drug repurposing offers an attractive strategy, leveraging existing compounds with known safety profiles. Selective serotonin reuptake inhibitors (SSRIs), primarily prescribed for psychiatric disorders due to their effects on central serotonergic signalling, have garnered increasing attention for their diverse peripheral effects, including modulation of immune and metabolic processes [14,15].

We conducted this study to assess the therapeutic efficiency of fluoxetine as adjuvant therapy in the treatment of severe sepsis and its effect on multiple organ dysfunction and mortality in the Intensive Care Unit (ICU).

Our study found that while the number of patients requiring vasopressors was lower in the fluoxetine group (39% vs. 61%, p = 0.14), this difference wasn’t statistically significant. However, the duration of vasopressor use was significantly shorter in the fluoxetine group (6.22 ± 0.44 days vs. 7.93 ± 0.83 days, p < 0.001). Comparison with Other Studies.

This finding aligns with research on the benefits of early intervention in septic shock. A study demonstrated that early hydrocortisone administration reduced vasopressor duration and ICU stay. Similarly, fluoxetine’s anti-inflammatory properties may contribute to decreased vasopressor needs [14].

Potential Mechanisms: Fluoxetine’s effects on vascular tone and response to vasopressors may be attributed to: Serotonin modulation: Influencing serotonin levels to affect vascular tone. Inflammation reduction: Anti-inflammatory properties that improve outcomes in sepsis [16]. Our study suggests that fluoxetine may reduce vasopressor needs in sepsis via anti-inflammatory and vascular effects, consistent with existing research on the topic. And this associated with significant reduction in ICU length of stay in fluoxetine group 15.91 ± 1.56 days versus 17.09 ± 1.12 in control group with p value: 0.005 and go with A study carried by Guinot and his colleague demonstrated that implementing vasopressor sparing strategies is associated with lower morbidity and ICU (intensive care unit) length of stays [17].

Vasopressor duration is a widely accepted and clinically relevant endpoint in sepsis trials, especially those investigating interventions targeting hemodynamic stability or inflammation [18]. Prolonged vasopressor use is associated with increased risk of adverse events, including ischemic complications, arrhythmia, and secondary infections. Thus, reducing vasopressor dependency is considered a marker of improved cardiovascular function and recovery from shock. Previous landmark sepsis studies (e.g., the VASST and ADRENAL trials) [19,20] have used vasopressor duration or time to shock reversal as key secondary or exploratory endpoints. Shorter vasopressor duration correlates with improved microcirculatory function and, in some cohorts, with reduced ICU length of stay.

Our study revealed statistically significant differences between the fluoxetine and control groups in: Lactate levels (p = 0.008), APACHE II scores (p = 0.028), SOFA scores on day 7 and day 10 (p = 0.047). These findings align with research by Tejaswini et al., which demonstrated the prognostic value of serum lactate, APACHE II, and SOFA scores in sepsis patients [21]. Comparison with Existing Research Tejaswini’s study showed that Serum lactate and prognostic scores (APACHE II and SOFA) were significantly different between survivors and non-survivors. Serial lactate monitoring had similar diagnostic accuracy to traditional prognostic scoring systems. However, our study found that while fluoxetine reduced lactate levels and prognostic scores, this reduction did not translate to a significant decrease in mortality rates (p = 0.369). Our findings suggest that fluoxetine may have beneficial effects on certain clinical parameters, but its impact on mortality rates requires further investigation.

The significance of fluoxetine’s effects extends beyond simple immunomodulation to encompass critical metabolic adaptations during sepsis. Sepsis is often associated with profound metabolic dysregulation, including alterations in lipid metabolism and energy utilization, which contribute significantly to organ dysfunction, particularly cardiac failure [7,22]. The work by Gallant et al [7] mechanistically links fluoxetine-induced IL-10 elevation to protection against sepsis-induced metabolic derangements. Specifically, IL-10 was found to be necessary for preventing hypertriglyceridemia and mitigating adverse cardiac effects, such as impaired glucose oxidation, ectopic lipid accumulation (cardiac steatosis), and ventricular stretching. This suggests that fluoxetine facilitates protective metabolic reprogramming, potentially preserving cardiac function during the septic insult via an IL-10-dependent mechanism. This integrated immunometabolic defense highlights a sophisticated interplay where immune modulation directly influences metabolic outcomes, contributing to overall host resilience.

In septic patients, TNF-α is the first pro-inflammatory cytokine to be released, followed by IL-1, IL-6, and IL-8. TNF-α and IL-1 are particularly significant among the proinflammatory cytokines; they are biologically interconnected, act synergistically, and play a major role in the clinical manifestations of sepsis [23].

As regard inflammatory marker our study showed On day 7 showed statistically significant differences (lower in fluoxetine group versus control group) CRP level (p = 0.004), pro-calcitonin level (p = 0.008), TNF-α level (p = 0.000), IL-1 level (p = 0.000) score (p = 0.047) (**Table 2**) and reflected on significant difference on length of stay (15.91 ± 1.56) in fluoxetine group versus (17.09 ± 1.12) in control group and (p = 0.005).

Recent research has begun to unravel the non-serotonergic, host-directed mechanisms through which fluoxetine might exert its beneficial effects during sepsis. Studies indicate that fluoxetine can orchestrate complex immunometabolic defenses, influencing both inflammatory pathways and systemic metabolism in ways that promote survival and limit organ damage in preclinical sepsis models [23]. These findings point towards potential “off-target” effects of fluoxetine that could be therapeutically exploited.

Observational studies in COVID-19 patients, another condition marked by hyperinflammation, have hinted at potential benefits associated with SSRI use, including reduced risk of intubation or death [8,24].

Furthermore, SSRIs have been shown to protect against sepsis in animal models [25]. The mechanisms underlying these protective effects are unclear. SSRIs have been reported to have anti-inflammatory effects, which suggests they may protect against overwhelming inflammatory responses and cytokine storm [26–30].

### Limitations

This single-center exploratory trial has several limitations, including a small sample size that limits power, especially for mortality outcomes, and limited generalizability. Fluoxetine was given post-sepsis onset, unlike preclinical models, and long-term effects were not assessed. Pharmacokinetic variability, heterogeneous sepsis etiologies, and unmeasured key biomarkers like interleukin-10 further limit mechanistic insight. While fluoxetine showed favorable effects on inflammation and vasopressor duration, its safety in broader ICU populations and its impact on mortality remain uncertain, warranting validation in larger, multicenter trials.

## Conclusion

Fluoxetine, as a member of the SSRI, is a promising agent as adjuvant therapy in severe sepsis on certain clinical parameters (duration of vasopressor, length of stay in ICU, organ dysfunction, and inflammatory markers), but its impact on mortality rates requires further investigation.

## Supporting information

S1 File CONSORT_2025_editable_checklist. (DOCX)

S2 File CONSORT_2025_flow_diagram. (DOCX)

S3 File Final protocol. (PDF)

S4 File Supplement data. (XLSX)
