# Supplementary material for: Effect of fluoxetine on organ dysfunction and mortality in severe sepsis
Source: PLoS One. 2026 Jan 21;21(1):e0340669. doi: 10.1371/journal.pone.0340669 (PMC12822927; doi:10.1371/journal.pone.0340669)
Supplement: S2 File — (DOCX) [file pone.0340669.s002.docx]

**Figure 1: CONSORT 2025 Flow Diagram**

Flow diagram of the progress through the phases of a randomised trial of two groups (that is, enrolment, intervention allocation, follow-up, and data analysis)

Allocated to intervention (n=23 )

Received convention management of sepsis and placebo tablet

Discontinued intervention (give reasons) (n=0 )

Lost to follow-up for primary outcome (give reasons) (n= 0):

Excluded (n=16 )

Not meeting inclusion criteria (n=9 )

Declined to participate (n=5 )

Other reasons (n=2 )

Analysis

Analysed for primary outcome (n=23 )

Excluded from analysis (give reasons) (n= 0)

Discontinued intervention (give reasons) (n= 0)

Lost to follow-up for primary outcome (give reasons) (n= 0):

Randomised (n= 46)

Allocation

Follow-Up

Allocated to intervention (n=23 )

Received convention management of sepsis and fluoxetine tablet 4 mg

Enrolment

Assessed for eligibility (n=62 )

Analysed for primary outcome (n=23 )

Excluded from analysis (give reasons) (n=0 )
