# Supplementary material for: Effect of fluoxetine on organ dysfunction and mortality in severe sepsis
Source: PLoS One. 2026 Jan 21;21(1):e0340669. doi: 10.1371/journal.pone.0340669 (PMC12822927; doi:10.1371/journal.pone.0340669)
Supplement: S3 File — (PDF) [file pone.0340669.s003.pdf]

Title of the Protocol:

Effect of fluoxetine on Organ Dysfunction and Mortality in Severe Sepsis

Author: Islam Abdelaal Abdelmouty Taher

Degree: M.B.B.CH., M.Sc., M.D. Anesthesia, faculty of medicine, Ain Shams University

Author: Mohammed Abdelwareth Mohammed

Degree: M.B.B.CH., M.Sc., M.D. Anesthesia, faculty of medicine, Ain Shams University

Author: Farouk Kamal Eldin Abdelaziz

Degree: M.B.B.CH., M.Sc., M.D. Anesthesia, faculty of medicine, Ain Shams University

Auther: Amr Sobhy Abdelqawy

Degree: M.B.B.CH., M.Sc., M.D. Anesthesia, faculty of medicine, Ain Shams University

1. INTRODUCTION / REVIEW:

Both sepsis and septic shock represent a major growing global burden and a challenge for physicians because of their increasing incidence and great pathophysiological, molecular, genetic, and clinical complexity. The incidence of sepsis and septic shock has continuously increased since the first consensus definition (Sepsis-1) in 1991, reaching around 49 million cases of sepsis and 11 million sepsis-related deaths worldwide in 2017 **(Chiu and Legrand 2021**)

Severe sepsis is defined as sepsis associated with organ dysfunction, hypoperfusion, or hypotension. Perfusion abnormalities may include, but are not limited to, lactic acidosis, oliguria, or alteration in mental status. In 2016, the surviving sepsis campaign defined sepsis as a life-threatening organ dysfunction caused by a dysregulated host response to infection **(Singer et al., 2016).**

In septic patients, TNF-α is the first pro-inflammatory cytokine that is released, followed by IL-1, IL-6, and IL-8. TNF-α and IL-1 are the most important proinflammatory cytokines; they are biologically closely related, act synergistically, and are largely responsible for the clinical manifestation of sepsis **(Schulte et al., 2013).** Fluoxetine is an antidepressant that belongs to a group of medicines called serotonin selective reuptake inhibitors (SSRIs). It works by increasing the amount of a natural chemical called serotonin in the brain **(Roumestan et al., 2007).**

It is now recognized that SSRIs also have a wide range of peripheral effects including regulation of immune and metabolic **processes (Olguner Eker et al., 2017; Szałach et al., 2019)**. Furthermore, SSRIs have been shown to protect against sepsis in animal models (**Rosen et al., 2019**) and improve outcomes in patients infected with SARS-CoV-2 (**Reis et al., 2022**). The mechanisms underlying these protective effects are unclear. SSRIs have been reported to have anti-inflammatory effects, which suggests they may protect against overwhelming inflammatory responses and cytokine storm (**Durairaj et al., 2015; Tynan et al., 2012**). They have also been reported to regulate aspects of systemic metabolism that are dysregulated during sepsis and other inflammatory states including lipid metabolism (**Chiu et al., 2021; Pan et al., 2018; Rozenblit-Susan et al.,2016**).

2. AIM OF THE STUDY:

The aim of this study is to evaluate the efficiency of fluoxetine as an adjuvant therapy in the treatment of severe sepsis and its effect on multiple organ dysfunction and mortality in septic patients.

3. METHODS:

Ethical Considerations: The study will be conducted after taking approval from the Research Ethics Committee at Ain-Shams University Hospital, Cairo, Egypt. An informed written consent will be obtained from each participant's legal guardian. Confidentiality of the data will be preserved.

Study Design: Randomized, controlled, double-blind study.

Study Setting and Duration: This study will be carried out at the Anesthesiology Department of Ain-Shams University Hospital, Cairo, Egypt from December 2024 to June 2025.

Patients and Methods:

Study Population:

- ASA II, III 60 patients, aged 18-65 years, BMI 30-35, admitted to ICU and diagnosed as patients with severe sepsis.
- The diagnosis of severe sepsis was established according to the definitions of the Surviving Sepsis Campaign (SSC) **(Singer et al., 2016).**

Severe sepsis definition = sepsis-induced tissue hypoperfusion or organ dysfunction (any of the following thought to be due to the infection)
Sepsis-induced hypotension
Lactate above upper limits laboratory normal
Urine output < 0.5 mL/kg/hr for more than 2 hrs despite adequate fluid resuscitation
Acute lung injury with $PaO_2/FIO_2 < 250$ in the absence of pneumonia as infection source
Acute lung injury with $PaO_2/FIO_2 < 200$ in the presence of pneumonia as infection source
Creatinine > 2.0 mg/dL (176.8 μmol/L)
Bilirubin > 2 mg/dL (34.2 μmol/L)
Platelet count < 100,000 μL
Coagulopathy (international normalized ratio > 1.5)

Exclusion Criteria:

- Patients with Late septic shock at presentation with multiple organ failure
- APACHE II score on admission >25
- Preexisting liver cirrhosis
- patients with prolonged QT interval or premature ventricular beats
- Preexisting end-stage renal disease
- Pregnancy
- History of allergy to fluoxetine
- Patients with malignancies
- Uncontrolled hemorrhage
- Burns
- Previous steroid therapy
- Death within 24 hours of ICU admission
- Concurrent use of MAO inhibitors or linezolid or any drugs increase QR interval like flecainde
- History of seizures
- Age more than 65 years

Patients will be recruited after admission to the Ain Shams University Hospital. Randomization will be performed using computer-generated random number tables in opaque sealed envelopes prepared by an anesthesiologist who will not participate in the study. Patients will be randomly divided into two groups using a computer-generated random number chart:

- **Group 1:** Receiving conventional sepsis therapy alone according

  to our hospital protocol (summarized as the following): -
- Initial Resuscitation:

- Patients with sepsis-induced tissue hypoperfusion with suspicion of hypovolemia to achieve a minimum of 30 mL/kg of crystalloids.
- The goals of initial resuscitation of sepsis-induced hypoperfusion included all of the following:
- MAP ≥ 65 mm Hg.
- Urine output ≥ 0.5 ml/kg/hr.
- CVP 8–12 mm Hg.
- Central venous oxygen saturation (ScvO2) 70%.
- <u>Diagnosis</u>
- Cultures including blood cultures before antimicrobial therapy if no delay in the start of antimicrobial.
- <u>Antimicrobial therapy</u>
- Iv broad spectrum (one or more anti-infective drug) to cover all likely pathogen according to site of infection and hospital antibiogram.
- <u>Source control</u>
- Source of sepsis should be identified, and intervention should be taken as soon as possible to control it (e.g. surgical drainage and change of infected lines)
- <u>Vasopressor</u>
- If adequate fluid resuscitation therapy was not able to restore hemodynamic, Vasopressor therapy initiated to target a mean arterial pressure (MAP) of 65 mm Hg.
- Norepinephrine as the first-choice vasopressor, epinephrine added to and potentially substituted for norepinephrine when an additional agent is needed to maintain adequate blood pressure
- <u>Corticosteroids</u>
- If adequate fluid resuscitation and vasopressor therapy are not able to restore hemodynamic stability, IV hydrocortisone at a dose of 200 mg/day.

- **Group 2:** Receiving conventional sepsis therapy in addition to fluoxetine 40 mg (Prozac 20mg , Elli lilly,s medical company Indianapolis, Indiana, United States) ® (1) daily for 28 days or until discharge from the ICU or death.
- **Primary outcome:** vasopressor dose.
- **Secondary outcome:** ICU Mortality & Organ dysfunction.

Procedures:
Clinical Evaluation:

- History and physical examination with special emphasis on vital signs (Blood pressure, pulse, Temperature, Respiratory Rate) and Glasgow Coma Scale to be continuously evaluated. drug to interaction will be considered when addi new drugs like the amino acids L-dopa and L-tryptophan, anorexiants, anticonvulsants, antidepressants, anxiolytics, calcium channel blockers, cyproheptadine, lithium salts, and drugs of abuse.
- Basl line ECG and calculation of QR interval and detection of ant type of arrythemia by cardiologist then follow up every otherday
- 10 ml blood sample will be taken there will be no leftovers and no sample storage after research ends and the following labartory parameters will be measursed

Routine Laboratory Investigations:

- CBC (Complete blood count)
- Coagulation profile: PT, INR, PTT
- ABGs (Arterial Blood Gases)
- Central Venous Oxygen Saturation
- Liver Function Tests: ALT (Alanine aminotransferase), AST (Aspartate aminotransferase), TBL (Total bilirubin), albumin
- Kidney Function Tests: urea, serum creatinine
- These routine labs will be withdrawn daily.

Labs Specific for the Study:

- Lactate
- CRP (C-reactive protein)
- Procalcitonin
- On days 1, 3, 7, 10, and 14.

Microbiological Studies:

- Including cultures (sputum, blood, urine or biological fluid according to clinical suspicion) on day 1.

Imaging Studies:

- Required to identify the source of sepsis (e.g., pelvi-abdominal CT, ultrasound, chest x-ray).

Clinical Data:

- To be evaluated up to a maximum follow-up period of 28 days of ICU stay:
    - Length of hospital stay
    - Need for and duration of mechanical ventilation
    - Need for and duration of vasopressor and inotropic support
    - Need for hemodialysis
    - Final outcome:
        - Survivors:
            - A) Patients recovered and discharged from ICU.
            - B) Patients still morbid and stayed in ICU more than 28 days.
        - Non-survivors:
            - Death before the completion of the 28 days of study period.

Application of APACHE II and SOFA Scoring Systems:

- APACHE II score to be evaluated on study day 1.

- SOFA score to be evaluated on study day 1 and serially every day until ICU discharge or death or up to 28 days.

Statistical Analysis and Sample Size:

- Data entry, processing, and statistical analysis will be carried out using SPSS v29. Frequency tables with percentages will be used for categorical variables and descriptive statistics (mean ± standard deviation or median with interquartile range) will be used for numerical variables. Tests of significance (Chi-square, student's t-test, or Mann Whitney's test) will be used according to the normality of the data. A P-value of less than 0.05 will be considered statistically significant. Total sample size 52 cases (organ dysfunction using SOFA score will be used as a primary outcome with proposed large effect size (0.8) and alfa=0.05 and power=0.80, so, 26 cases are needed in each group).

Sample Size Calculation:

- The sample size was based on a meta-analysis by Rygård et al. (9), which reported a mean reduction in shock duration of −1.52 days with corticosteroids compared to placebo (95% confidence interval: −1.71 to −1.32). Using this effect size, a two-sided alpha of 0.05, and 80% power, we calculated that 23 patients per group (46 total) would be sufficient to detect a statistically significant difference.

Reference: **Rygård SL, Butler E, Granholm A, Møller MH, Cohen J, Finfer S, Perner A, Myburgh J, Venkatesh B, Delaney A**. Low-dose corticosteroids for adult patients with septic shock: a systematic review with meta-analysis and trial sequential analysis. Intensive Care Med. 2018 Jul;44(7):1003-1016. doi: 10.1007/s00134-018-5197-6. Epub 2018 May 14. PMID: 29761216.

Statistical Analysis:

- Results will be expressed as mean ± standard deviation or number (%). Comparison between categorical data will be performed using the Chi-square test. Comparison between different numerical data in the two studied groups will be performed using an unpaired t-test. Comparison between different times of measurements and baseline within the same group will be performed using repeated measure ANOVA. The SPSS computer program will be used for data analysis. P value less than 0.05 will be considered significant.

4. REFERENCES:

(1) medscape.com/drug/prozac-fluoxetine-342955

Adhikari NK, Fowler RA, Bhagwanjee S, Rubenfeld GD. (2010). Critical care and the global burden of critical illness in adults. The Lancet; 376(9749): 1339-1346.

Chiu C., Legrand M. Epidemiology of sepsis and septic shock. *Curr. Opin. Anaesthesiol.* 2021;34:71–76.

Chiu, Y.-J., Tu, H.-H., Kung, M.-L., Wu, H.-J., Chen, Y.-W., 2021. Fluoxetine ameliorates high-fat diet-induced metabolic abnormalities partially via reduced adipose triglyceride lipase-mediated adipocyte lipolysis.Biomed. Pharmacother. 141, 111848. https://doi.org/10.1016/j.biopha.2021.111848

Dellinger RP, Levy MM, Rhodes A, Annane D, Zimmerman JL. (2013). Surviving sepsis campaign: International guidelines for management of severe sepsis and septic shock: 2012. Critical Care Medicine; 41(2): 580–637.

Durairaj H, Steury MD. (2015). Antidepressants to disrupt inflammation: Considering fluoxetine as an alternative to anti-inflammatory treatments for depression. Neuropsychiatric Disease and Treatment; 11:1793-1800.

Evans L., Rhodes A., Alhazzani W., Antonelli M., Coopersmith C.M., French C., Machado F.R., Mcintyre L., Ostermann M., Prescott H.C., et al. Surviving sepsis campaign: International guidelines for management of sepsis and septic shock 2021. *Intensive Care Med.* 2021;47:1181–1247. doi: 10.1007/s00134-021-06506-y.

Olguner Eker, O., Ozsoy, S., Eker, B., Dogan, H:Metabolic Effects of Antidepressant Treatment. Arch Neuropsychiatry 54, 49‑56. https://doi.org/10.5152/npa.2016.12373.

Pan, S., Tan, Y., Yao, S., Xin, Y., Yang, X., Liu, J., Xiong, J., 2018. Fluoxetine induces lipid metabolism abnormalities by acting on the liver in patients and mice with depression. Acta Pharmacol. Sin. 39, 1463‑1472. https://doi.org/10.1038/aps.2017.207

Rygård SL, Butler E, Granholm A, Møller MH, Cohen J, Finfer S, Perner A, Myburgh J, Venkatesh B, Delaney A. Low-dose corticosteroids for adult patients with septic shock: a systematic review with meta-analysis and trial sequential analysis. Intensive Care Med. 2018 Jul;44(7):1003-1016. doi: 10.1007/s00134-018-5197-6. Epub 2018 May 14. PMID: 29761216.

Roumestan C, Michel A, Bichon F, Portet K, Detoc M, Henriquet C, Jaffuel D, Mathieu M. (2007). Anti-inflammatory properties of desipramine and fluoxetine. Respir Res; 8(1):35. DOI: 10.1186/1465-9921-8-35.

Rosen D, Sneed KB, Moses BK, et al. (2019). The Neuroprotective Effects of Fluoxetine in Septic Shock. Journal of Critical Care; 53:98-109.

Reis G, Silva EA, Silva DC, et al. (2022). Effect of early treatment with fluoxetine on risk of hospitalization among patients with COVID-19: A randomized clinical trial. JAMA; 327(6): 547-553.

Rozenblit-Susan, S., Chapnik, N., Froy, O., 2016. Metabolic effect of fluvoxamine in mouse peripheral tissues. Mol. Cell. Endocrinol. 424, 12–22. https://doi.org/10.1016/j.mce.2016.01.009.

Singer M, Deutschman CS, Seymour CW, Shankar-Hari M, Annane D, Bauer M, Bellomo R, Bernard GR, Chiche JD, Coopersmith CM, Hotchkiss RS. (2016). The third international consensus definitions for sepsis and septic shock (Sepsis-3). JAMA; 315(8): 801-810.

Schulte W, Bernhagen J, Bucala R. (2013). Cytokines in Sepsis: Potent Immunoregulators and Potential Therapeutic Targets—An Updated View. Mediators of Inflammation; Article ID 165974, 16 pages.

Szałach, Ł.P., Lisowska, K.A., Cubała, W.J., 2019. The Influence of Antidepressants on the Immune System. Arch. Immunol. Ther. Exp. (Warsz.) 67, 143–151. https://doi.org/10.1007/s00005-019-00543-8

Tynan RJ, Naicker S, Wolvetang EJ, et al. (2012). Chronic stress-induced regulation of microglia and IL-1beta: implications for stress-related CNS illnesses. Neurosci Biobehav Rev; 36(6):1647-1659.
